# Initial COVID-19 Outbreak: An Epidemiological and Socioeconomic Case Review of Iran

**DOI:** 10.3390/ijerph17249593

**Published:** 2020-12-21

**Authors:** Elise Blandenier, Zahra Habibi, Timokleia Kousi, Paolo Sestito, Antoine Flahault, Liudmila Rozanova

**Affiliations:** 1 Department of Global Health, Global Studies Institute, University of Geneva, 1211 Geneva, Switzerland; elise.blandenier@etu.unige.ch (E.B.); timokleia.kousi@etu.unige.ch (T.K.); paolo.sestito@etu.unige.ch (P.S.); 2 Faculty of Medicine, Institute of Global Health, University of Geneva, 1211 Geneva, Switzerland; antoine.flahault@unige.ch; 3 Global Studies Institute, University of Geneva, 1211 Geneva, Switzerland; liudmila.rozanova@unige.ch

**Keywords:** Iran, COVID-19, crisis management, mathematical modeling, political trust crisis, pandemic, economy

## Abstract

The coronavirus disease 2019 (COVID-19) pandemic has affected millions of people worldwide. It brought about the implementation of various measures and restrictions at a global level. Iran has been one of the countries with the highest rates of COVID-19 cases. This study reviews the initial outbreak of COVID-19 in Iran and examines the mitigation strategies adopted by the country. Moreover, it reports the socioeconomic challenges faced by the authorities during the efforts to contain the virus. A transdisciplinary literature review was carried out and a political measures timeline was constructed. A broad overview of the initial phase of the COVID-19 outbreak in Iran is presented, starting from the first confirmed case on 19 February 2020 until April 2020. The results of this epidemiological and socioeconomic case review of Iran suggests that the political measures undertaken by the Republic of Iran contributed to the decrease of the prevalence of COVID-19. However, due to the existing financial bottleneck, Iran has faced limited health system resources. Therefore, the response was not sufficient to restrict the spread and the efficient handling of the virus in the long-term.

## 1. Introduction

In early December 2019, a local outbreak of pneumonia was detected in Wuhan city of Hubei Province, China. This outbreak was later known to be caused by a novel coronavirus, severe acute respiratory syndrome coronavirus 2 (SARS-CoV-2) [1]. The family of coronaviruses to which this RNA virus belongs can cause respiratory tract infections of various severities. These infections range from cases of the common cold to more serious conditions [2]. On 11 March 2020, the Director General of the World Health Organization declared SARS-CoV-2 as a pandemic [3]. In March 2020, the Islamic Republic of Iran was among the nations with the highest rate of cases [4]. Like many other countries, Iran was hit hard by the virulence of the virus. Iran’s first official coronavirus disease 2019 (COVID-19) case was recorded on 19 February 2020 in Qom [5]. Given that Iran was also the first country in the Middle East where the virus was detected, it is possible that it played a key role in the dissemination of the disease in neighboring countries, such as Iraq, Pakistan, and Afghanistan [6]. The epidemiological situation of Iran and the complexity of the political and economic challenges of the country make this country a particularly relevant subject of study.

This study is divided into different subsections to thoroughly explore Iran’s case; the epidemiological situation in the country, pharmaceutical and non-pharmaceutical measures adopted, mathematical modeling, the economic and political context of the country, and finally the consequences of these characteristics on the expansion of the COVID-19 outbreak.

## 2. Methods

This observational study is conducted based on real-time information reported by different sources, such as official websites of authorities of Iran, national and provincial health agencies, published articles, higher educational institutions, and the news agencies between February 2020 and April 2020. Although the information regarding the outbreak of COVID-19 is still continuously evolving, all of the data provided in this case study represents the initial outbreak of the pandemic in the Islamic Republic of Iran at the time of the study publication. All information used for this study was publicly available and, thus, no ethics approval was required. In addition, the gathered information was derived from accredited and reliable sources.

The mathematical modeling part of this study was conducted based on the Susceptible, Exposed, Infected, Removed (SEIR), a common mathematical model usually used to study the transmission of a disease in research studies. Apart from the spread of the virus, the current simulation allows us to investigate the burden for the healthcare system and the expected number of deaths in various scenarios [7].

Lastly, this case review is a real-time analysis of the initial COVID-19 outbreak in Iran since the information from the event is taken as the pandemic was still unfolding. Therefore, most of the data and possible outcomes continue to evolve.

## 3. Country’s Overview and Results

### 3.1. Case Presentation

#### 3.1.1. Demographic, Economic, Geographic, Political, and Climatic Characteristics of Iran

Iran, officially “the Islamic Republic of Iran” [8], is a country located in the Middle East and Western Asia [8]. It is the 18th largest country in the world, covering an expanse of 1.628–750 sq km^2^, with a population of 83.70 million in 2020 [9]. The number of adults in the country is estimated around 59.163 million and the population is increasing at a rate of 1.36 in 2020 [10]. Despite the large landmass, much of the land is uninhabitable, and is associated with a population density of 51.6 people per sq km [8]. The country has a subtropical climate influenced by the subtropical drought of the Arabian Desert, and the humidity of the Mediterranean region. For most of the year, the weather is warm, and the temperature is above 25 degrees [11].

From a political perspective, Iran is an Islamic republic based on the 1979 constitution [12]. The country is governed by a mixed system, where the executive, legislative, and judiciary are being superintended by several bodies composed mainly of clergy [9]. The supreme leader, known as the Rahbar, comes from the clergy, and has the highest power in the country [13].

From an economic point of view, Iran’s Gross Domestic Product (GDP) was estimated around US $447.7 billion in 2017. Iran has the second-largest natural gas reserves and fourth crude oil reserves globally. As a result, the government and economy depend largely on oil. Other noticeable economic sectors are agriculture and service sectors, as well as manufacturing and financial services [14].

#### 3.1.2. Healthcare System

The healthcare system in Iran is considered quite effective. For more than two decades, the government has emphasized that the primary healthcare services, and some specialized medical procedures, such as prenatal care and vaccinations, are free of charge [15]. Even though the private sector plays a significant role in the health system in Iran, most of the secondary and tertiary health services are provided by the public sector [15]. According to Letafat et al. (2018) [16], there are no proper policies that are aimed to harmonize the insurance system. In the same report, they also noted the limitation of financial resources and lack of attention to cost management, and lack of using evidence-based medicine for effective interventions. Funding regarding the health system remained an issue in Iran. It is also important to underline that the health system in Iran is currently reformed towards Iran’s vision 2025 and Iran’s Health Innovation and Science Development Plan [16]. Moreover, according to recent studies, Iran’s healthcare system is getting closer to the World Health Organization (WHO)–Universal Health Coverage (UHC) recommendations [16]. However, some impediments remained, such as inadequate budgets, lack of clear borders between public and private systems, multiplicity of insurance organizations and insurance funds, drastic changes in epidemiology of diseases and demographic characteristics, lack of accountability to demands of society due to the limitations of manpower employed in the health sector, and negligence of social variables in this sense [16,17,18].

According to the WHO and International Health Regulation (IHR) state-party self-assessment report from 2017, Iran considered themselves as perfectly ready to manage an emergency health response operation with a score of 100% [19].

#### 3.1.3. Epidemiological Situation of Iran Regarding COVID-19

**Figure 1:**
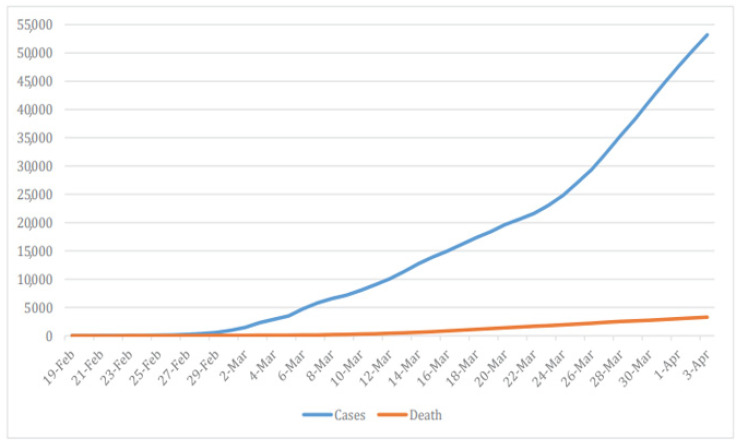
Daily number of deaths and daily number of cases since the beginning of the outbreak in Iran. Reproduced from [4], Copyright 2020, Raoofi, A.; Takian, A.; Sari, A.A.; Olyaeemanesh, A.; Haghighi, H.; Aarabi, M.

As of 28 March, Iran reported 35,408 confirmed cases, 2517 total associated deaths, and 11,679 total recovered cases [20], Figure 1. Therefore, there were 423.03 cases per million population and 30.07 deaths per million population confirmed in the country [21]. At this time, Iran ranked 7th in the list of the number of confirmed cases globally [22]. The Case Fatality Rate (CFR) was estimated around 7.5%, twice as high as the global average, with only Italy showing a higher rate [23].

**Figure 2:**
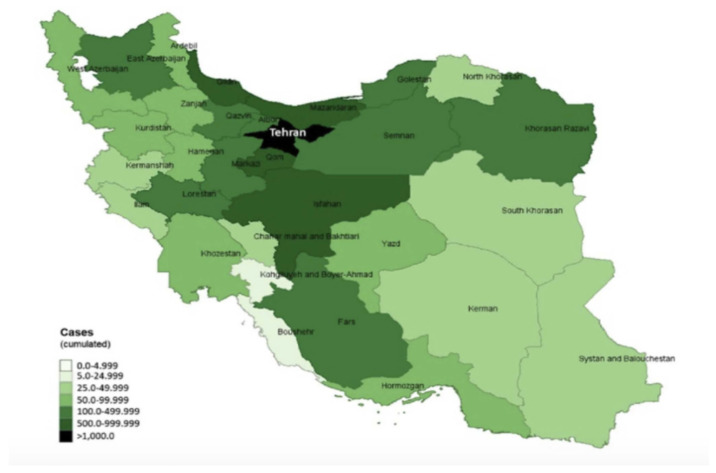
Case distribution by provinces, 9 March 2020. Reproduced from [6], Copyright 2020, Arab-Mazar, Z.; Sah, R.; Rabaan, A.A.; Dhama, K.; Rodriguez-Morales, A.J.

The first two cases were announced on 19 February 2020, in the city of Qom. After this, the virus spread quickly in neighboring areas, such as Tehran, Markazi, Isfahan, and Semnan provinces, Figure 2. As of 9 March 2020, the cities with the highest number of confirmed cases were Tehran (1945), Qom (712), and Isfahan (601) [6,24]. Despite the increase of numbers in Tehran, when considering the local population, we can compute that Qom still has the highest number of cases, with 791.11 cases per million population; Isfahan is second with 388.45 cases per million population, and Tehran is last with 271.90 [25]. A little while later, the disease spread to all provinces in the country [26]. The daily incidence increased rapidly; on 30 March 2020, there was a total of 3186 new confirmed cases [27].

The city with the highest number of confirmed cases was Tehran (1945) followed by Qom (712) and Mazandaran (633), as of 19 March 2020 [28]. In addition, the Deputy Minister of Health announced that, as of 14 March, the male to female ratio was 1.4, and the mean age of patients was 54 years of age. The mean age of the patients that did not survive was 64 years of age. He added that the percentage of patients who died and suffered from underlying diseases was 43.7%. In addition, the most common symptoms at the time of admission were fever and cough (54% of the cases) [29].

There are concerns that the actual burden of disease in Iran was greater than what was reported [29]. Dr. Brennan, the Director of Emergency Operations in WHO, after his mission in Iran, announced that the number of confirmed cases represent only a fraction of the real cases, due to lack of testing [30]. The confirmed number of deaths is also questionable as there are rumors that deaths due to COVID-19 are underreported in death certificates [31].

By 31 May 2020, Iran reported a cumulative total of 151,466 confirmed cases and 7797 deaths. Despite the decrease in the daily incidence that was apparent at the end of April 2020, the daily reported cases increased again from the beginning of May. More specifically, on 31 May 2020, Iran recorded daily incidence of 2516 [32].

### 3.2. Management and Outcome

#### 3.2.1. Non-Pharmaceutical Measures

Since the beginning of the outbreak in Iran, the Iranian government started undertaking strict non-pharmaceutical interventions in various aspects. Social media, television, and radio were used for raising awareness regarding preventive measures for COVID-19 in society. Announced by the Central Bank of Iran, the government started minimizing the use of currency notes during this outbreak [33]. To control the spread of the virus in prisons, more than 70,000 prisoners who were sentenced to less than five years for non-violent crimes were released in early March. The release followed a confirmation of negative COVID-19 tests [34].

In late February, the Iranian Ministry of Health established national and regional hotlines for COVID-19. Moreover, different websites were designed to track and refer people to healthcare centers by using a short questionnaire. These websites also provided information regarding the high-risk neighborhoods of the cities. The official website of Iran’s ministry of health for COVID-19 is “http://corona.behdasht.gov.ir”. Along with this source, there are other major websites such as one for screening for the disease via the Health Ministry’s online platform, “salamat.gov.ir”. An application for coronavirus, named AC19, released by Iranian government, was removed from Google Play, claiming that it was handling user data without respecting confidentiality [35].

Opening hours for tourist spots, including museums, palaces, and historical places were reduced, and eventually, with the sudden increase in cases at the end of March, most of these places closed. Starting from 1 March, all the arts and culture programs were suspended indefinitely [33].

According to the official website of the President of the Islamic Republic of Iran, on 19 March, a 15-day shutdown of all non-essential businesses and services in several provinces was announced. President Rouhani directed the Minister of Interior Affairs, on March 20, to close shopping malls and markets until 3 April [36]. Furthermore, twenty percent of the year’s budget was pledged to be allocated to COVID-19 response, about 1,000,000 Iranian Rial (IRR). A part of this allocation was dedicated to health and unemployment insurance, claiming that employers who kept their workforce would be given low-interest loans [37].

Moreover, government and insurance companies announced that, for patients whose COVID-19 test results were positive, a minimum of 90% of the total cost of their treatments would be covered, and all additional costs would be calculated based on public prices [37].

Working hours were reduced in some industries, and in February, schools and universities closed until the end of Nowruz holidays, on 3 April [33]. The starting date of school closures depended on the prevalence of the disease in each city. These closures led to a higher number of travelers during Nowruz, which increased the risk of spreading the virus in popular tourist cities, such as Isfahan. Therefore, numerous provincial officials emphasized this threat and asked people to avoid traveling [34] At that time, concerns regarding the spread of the virus exacerbated, as strict preventive measures, such as road closures or mandatory quarantine policies, were not applied in the country [38].

On 25 March, the National COVID-19 Administration in Iran announced stricter restrictions to control the spread of the disease. Iran imposed a travel ban at the end of March. According to government representatives, travel between cities was to be stopped. It was then prohibited for Iranian citizens to start new trips and leave cities [37]. In addition, the major streets of Tehran were disinfected repeatedly by Tehran’s Fire Department before and during the New Year holidays in March [39]. People were identified as residents of a city, with proof, such as a national code, car number, and car insurance. A “traffic card” was issued for workers who had to travel between cities. The restrictions were applied from 27 March until 3 April [40]. Furthermore, on 26 March, stricter rules were imposed on those who did not comply with health recommendations. If cars violated these new regulations, the cars were confiscated, for up to one month, and fined 5,000,000 IRR [41].

In addition, different guilds were sealed for the month of March. Violations of these rules resulted in fines, such as car seizures and trade union seals. The guilds which met the daily needs of the people, continued working, but there was a list of businesses to close. Moreover, governors had the power to close parks, sports centers, and other places where groups of people could gather [41].

#### 3.2.2. Political and Social Issues

The COVID crisis appears in an already very troubled time in Iran, following U.S. sanctions. Since the beginning of the pandemic, the government had been accused of a lack of transparency. The Non-Governmental Organization “Reporters sans Frontières” claimed that journalists had been harassed and the Iranian government was obscuring the reality of the spread of the virus. According to the scientific journal *Foreign Affairs*, the public asked for strong restrictions, but the authorities were slow to react. The media added that the Iranian bureaucracy showed itself unable to respond in a coherent and effective manner and, hence, the government lost the already low public trust [42]. In January 2020, the government hid, for three days, the fall of the Ukrainian plane shot down by the Pasdaran [43]. Events such as this exacerbate the (already existing) lack of public confidence, not only in political authorities, but also in the media [44]. National media, including the Islamic Republic of Iran Broadcasting (IRIB), lack credibility. As a result, fearing misinformation and the lack of transparency, the public turns to social media. This results in cure rumors, and incidents, such as the one that killed (at the beginning of March) 44 people who drank adulterated alcohol, in order to get rid of the virus, because of a rumor that circulated on the internet [45]. Without public trust, the Iranian government is going to struggle even more to tackle the pandemic [5]. It seems that they are already aware of this issue, as their WHO and IHR State-Party self-assessment report from 2017 identified the need to improve communication with the public (self-aware score: 43%) [19].

The Iranian political line, and the historical context of the country, make the government sometimes suspicious about external help coming from the international community. At the end of March, Iran first refused the medical and logistical help offered by Médecin Sans Frontières (MSF) in the city of Isfahan. The authorities claimed that MSF had underestimated the Iranian medical capacities in this city [46]. However, the decision was the source of many discussions. The Iranian Ministry of Health declared that they were going to accept from now on all external help, except from the U.S. and Israel. This case illustrated the entanglement between internal and external affairs in context of the pandemic [44].

The complexity of Iranian politics, closely interwoven with religious authorities, makes the management of the crisis even more delicate. It seems that the source of the virus spread in Iran is the city of Qom, a holy city that welcomes thousands of tourists every year. According to the Lebanese newspaper, L’Orient Le Jour, the city’s religious authorities strongly opposed the quarantine of the city [47].

Moreover, Iranian authorities feared the repercussions of Nowruz, the Iranian New Year. This occasion caused a very large displacement of the population, leading to possible consequences (the spread of the virus in the country) [48]. According to Karim Hemmati, the Iranian Red Crescent Society (IRCS) chief, nearly 3 million people from the 13 provinces involved in the COVID-19 pandemic, were traveling during Nowruz holidays, by 21 March [48]. Due to misconceptions about COVID-19, people were traveling in the country while Iranian authorities and the World Health Organization repeatedly urged them to stay home to prevent the spread of COVID-19 [49]. One noticeable message in this scenario is that people (citizens) are in charge of taking the necessary precautions into account—they are the ones who should actively follow the given guidelines [50]. A lack of self-care and disrespect for preventive measurements could be a result of insufficient awareness among different parts of society [42]. This emphasizes the importance of improving health literacy and health awareness among the societies [42]. During the spread of infectious diseases, such as the COVID-19 pandemic, it is the citizens who play a critical role in controlling the spread of the disease by following the preventive measurements and working as a harmonic team [42].

Most of the policies implemented from the beginning of April to the beginning of May, even if some were not completely accepted by the population, were successful in containing the spreading of the virus. Indeed, the prevalence of the disease decreased significantly [51]. However, the ease of social distancing measures was misconceived by the population. As a result, Iran observed an increase in the number of cases from the beginning of May, signaling a second wave [51].

#### 3.2.3. Economic Impact

Investors are concerned that the spread of COVID-19 could lead the global economy into a recession, which could bring an end to the longest economic expansion in history [52]. In the case of Iran, the negative economic impact is particularly noticeable. In early 2020, the country gradually began to recover from U.S. sanctions, which came into force in November 2018 [53]. As a result of these sanctions, the government of Iran lost at least 40% of its budget revenue [54]. In 2020, due to the overlap of U.S. sanctions and COVID-19, Iran is facing yet another severe economic challenge [54]. The country still finds itself deprived of a fully intact health system due to the financial constraints coming from the unilateral sanctions imposed by the U.S. [55,56]. It lacks the resources to provide sufficient diagnostic and pharmaceutical tools, as well as laboratory equipment, to efficiently combat COVID-19 [55].

After the rapid rise in COVID-19 infections in Iran in late February 2020, Iraq, and Turkey, two of the most important Iranian export markets, began a cancelation of flights and closed the borders with Iran [53]. Subsequently, more and more countries began to restrict free movement of people and goods with Iran. Apart from China as a central trading partner, almost all flight connections to Iran have been canceled [53]. Hence, the limited market activity is a challenge for many private companies. In particular, small and medium-sized companies (SMEs) are at risk of bankruptcy. However, due to the weakened financial system caused by the sanctions, Iran will not be capable of supporting and saving most of Iranian SMEs through financial aid packages [54]. However, Keogh-Brown and Smith (2008) observed that, during the Severe Acute Respiratory Syndrome (SARS) outbreak in 2003, it was rather difficult to derive accurate predictions of the macroeconomic impact on the country during an outbreak. The macroeconomic impact of SARS in 2003 turned out to be much smaller than predicted by model estimates and media reports [57]. Nevertheless, it should be considered that there are countries in economic distress (e.g., Iran) that will suffer the most in terms of health and economic prosperity [53,55]. In retrospect, looking at the first COVID-19 wave, evidence suggests that Iran’s financial resources were not sufficient to subsidize companies during the lockdown [57]. This led to continued business activity, making social distancing, and controlling the spread of the virus, unfeasible [57,58,59].

#### 3.2.4. Mathematical Modeling Prediction

Mathematical modeling, providing future projections regarding the COVID-19 outbreak in Iran, was created with the Epidemic Calculator Goh, which is based on a SEIR model (Susceptible-Exposed-Infectious-Removed model). The presented simulation divides I and R into mild cases that do not need hospitalization; moderate cases that are hospitalized and fatal, and that need hospitalization, but do not survive [7]. “I” stands for the number of initial infections and “R” is a measure of contagiousness, representing the number of secondary infections attributed to one specific individual. The simulation assumes that the country population and the rate of infections remain constant. In addition, for simplicity, it assumes that all fatalities happen in the hospital and all fatal cases are hospitalized right after the infectious period. The diagram presents the estimated number of people who are actively infectious per day, the people who are exposed to the virus, as well as the hospitalized patients and the total number of deaths. People who recovered are not presented in the diagrams.

**Figure 3:**
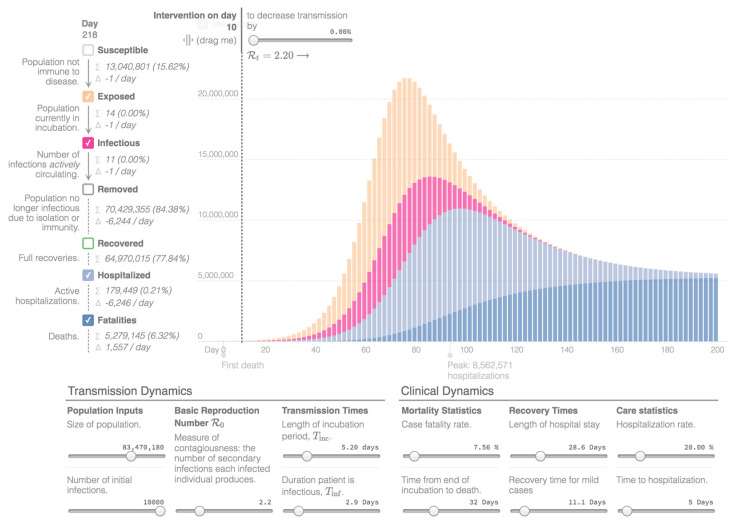
Epidemiological Curve 1 [7].

**Figure 4:**
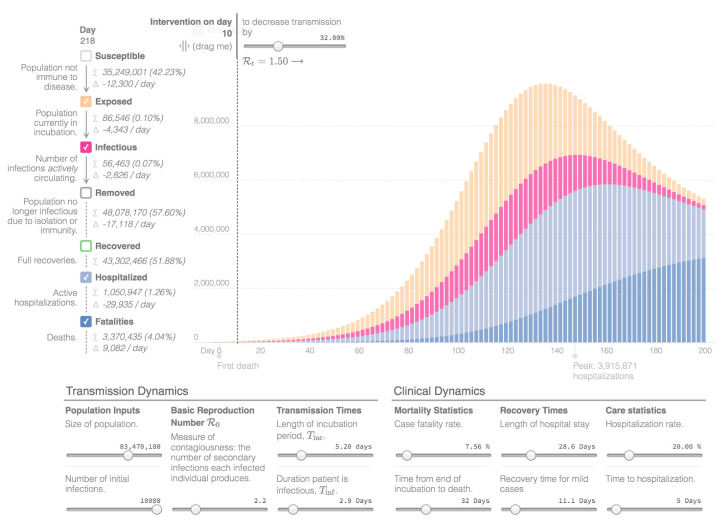
Epidemiological Curve 2 [7].

**Figure 5:**
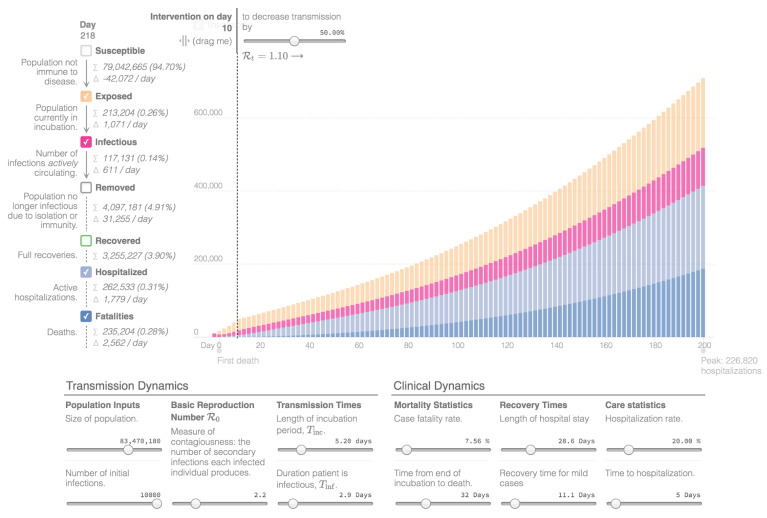
Epidemiological Curve 3 [7].

For the presented simulations, day 0 is 12 March with 10,000 confirmed cases. The total population of the country is estimated around 83 million [21] and the basic reproduction number (R0) is estimated at 2.2 [7,60]. The length of incubation time (Tinc) is estimated 5.2 days and the duration a patient is infectious (Tinf) is 2.9 days [7,60,61]. Regarding the clinical dynamics, the Crude Fatality Rate is estimated at 7.5% [22], the time from end of incubation to death is 32 days and the length of hospitalization 28.6 days (3 to 6 weeks), while the hospitalization rate is estimated at 20%, as is the estimate for serious and critical cases [7,60]. Furthermore, the recovery time for mild cases is estimated at 11.1 days (around 2 weeks) and the time for hospitalization 5 days [7,60]. The first epidemiological curve, Figure 3, shows the projection of the coronavirus outbreak in Iran without the implementation of measures. The second epidemiological curve, in Figure 4, reveals the projection after the implementation of measures on day 10 that decreases transmission by 32% (R0 = 1.54). The third epidemiological curve, in Figure 5, is the projection after the implementation of measures on day 10 that decreases transmission by 50% (R0 = 1.1). Considering the total number of cases and deaths on 31 May 2020, as reported above, which represents the 80th day of the diagrams, this implies that Iran managed to decrease the transmission of the virus by at least 50%.

## 4. Discussion

The COVID-19 pandemic has affected Iran since 19 February 2020, after the first case in the country was reported. Although Iran is a country that confronted epidemics in the past, COVID-19 has surprised the system by its magnitude, rapid spread, and consequences [62]. Iran’s economic and social situation, along with its healthcare system, has faced many new challenges since the beginning of the outbreak.

The Islamic Republic of Iran, on its territory, responded rapidly to the spread of the COVID-19 outbreak. Governmental measures may have not necessarily received strong public support, but the restrictive measures, subsequently put in place, did regain this public support [51]. The health measures have been effective in significantly reducing the prevalence of the virus in Iran.

However, it seems that the public would rather follow incorrect information coming from social media rather than governmental sources. This could lead to sanitary issues, as we demonstrate in our paper. On the other hand, it seems that the entanglement between religion and politics in Iran delayed some measures that could have been taken earlier, as was the case at Qom. These two factors should therefore be considered, and more effective communication policies should be adopted. Indeed, a population’s adherence to health measures is intrinsically linked to the quality of communication on the part of the authorities. All media vectors must be exploited to ensure the proper transmission of health information and to slow down the dissemination of erroneous and potentially dangerous information [63].

In addition, it is very likely that pre-existing inequalities among classes are going to worsen due to the economic impact of the outbreak. We, therefore, re-emphasize the importance of stratified data on this issue. The socioeconomic consequences of the pandemic are diverse and affect each population group differently. Pre-existing inequalities are reinforced by certain health measures [64]. It is therefore necessary for authorities to adopt a comprehensive approach and consider the different socioeconomic states when implementing health policies.

The end of the first wave should allow health authorities to implement the gains made. Without prophylaxis and a vaccine, a rapid recrudescence of the virus is highly likely. These new waves are problematic because they will occur in a complicated context: a weakened hospital environment, fragile economy, political tensions, and public skepticism or lassitude. They will therefore represent a real challenge for the Iranian authorities.

From the mathematical modeling of the outbreak, we can highlight the importance of the implementation and adaptation of prophylactic measurements. In case there are no measurement implementations, aiming to reduce the transmission of the disease, the number of cases and, consequently, the number of hospitalizations, might rise in an unsustainable way, regarding the limits of the healthcare system. In contrast, if society succeeds at maintaining a low transmission rate, the benefits, concerning the total number of hospitalizations and deaths, will be substantial.

## 5. Conclusions

Regarding the social and political context, this case review observed a substantial media coverage of the COVID-19 outbreak and found the measures taken by the Iranian government to be appropriate. However, there was a barrier, regarding the correct implementation of these measures, due to the mistrust between the public and authorities. In this context, it should be emphasized that Iran was already in a deprived economic state at the beginning of the pandemic [51,52]. Hence, because of the economic standstill caused by non-pharmaceutical measures, such as temporary closure of non-essential businesses, which poses as an additional economic challenge, serious economic consequences are to be expected in the near future. The public policies of the Islamic Republic of Iran carried out between February 2020 and the end of April 2020 were successful and decreased the number of COVID-19 cases [45]. However, Iran’s financial resources proved to be insufficient at subsidizing companies during the lockdown, which led to continued business activity leading to increased transmission rates of the virus [56,57].

As for the limitation of this study, there was a lack of scientific and stratified data to use by the time of writing the article. The spread of the virus has been extremely diversified; different regions of Iran are affected in different ways. Moreover, by the time of writing this article, it is difficult to estimate the results and the impacts of the outbreak. Obviously, we may have different results by the time the pandemic ends. Another study limitation is that we only focus on the first months of the COVID-19 outbreak in Iran (since the first reported case on 19 February 2020). It seems that it is early to assess the long-term effects of the virus in the country. Further research is needed to investigate the additional implemented measures and the potential future spread of the virus among the population.
